# Impact of a Wearable Device-Based Walking Programs in Rural Older Adults on Physical Activity and Health Outcomes: Cohort Study

**DOI:** 10.2196/11335

**Published:** 2018-11-21

**Authors:** Il-Young Jang, Hae Reong Kim, Eunju Lee, Hee-Won Jung, Hyelim Park, Seon-Hee Cheon, Young Soo Lee, Yu Rang Park

**Affiliations:** 1 Division of Geriatric Medicine Department of Internal Medicine Asan Medical Center Seoul Republic of Korea; 2 Department of Biomedical Systems Informatics Yonsei University College of Medicine Seoul Republic of Korea; 3 Graduate School of Medical Science And Engineering Korea Advanced Institute Of Science And Technology Daejeon Republic of Korea; 4 Pyeongchang Health Center & Country Hospital Gangwon-do Republic of Korea

**Keywords:** adherence, frailty, older adult, rural area, wearable device

## Abstract

**Background:**

Community-dwelling older adults living in rural areas are in a less favorable environment for health care compared with urban older adults. We believe that intermittent coaching through wearable devices can help optimize health care for older adults in medically limited environments.

**Objective:**

We aimed to evaluate whether a wearable device and mobile-based intermittent coaching or self-management could increase physical activity and health outcomes of small groups of older adults in rural areas.

**Methods:**

To address the above evaluation goal, we carried out the “Smart Walk” program, a health care model wherein a wearable device is used to promote self-exercise particularly among community-dwelling older adults managed by a community health center. We randomly selected older adults who had enrolled in a population-based, prospective cohort study of aging, the Aging Study of Pyeongchang Rural Area. The “Smart Walk” program was a 13-month program conducted from March 2017 to March 2018 and included 6 months of coaching, 1 month of rest, and 6 months of self-management. We evaluated differences in physical activity and health outcomes according to frailty status and conducted pre- and postanalyses of the Smart Walk program. We also performed intergroup analysis according to adherence of wearable devices.

**Results:**

We recruited 22 participants (11 robust and 11 prefrail older adults). The two groups were similar in most of the variables, except for age, frailty index, and Short Physical Performance Battery score associated with frailty criteria. After a 6-month coaching program, the prefrail group showed significant improvement in usual gait speed (mean 0.73 [SD 0.11] vs mean 0.96 [SD 0.27], *P*=.02), International Physical Activity Questionnaire scores in kcal (mean 2790.36 [SD 2224.62] vs mean 7589.72 [SD 4452.52], *P*=.01), and European Quality of Life-5 Dimensions score (mean 0.84 [SD 0.07] vs mean 0.90 [SD 0.07], *P*=.02), although no significant improvement was found in the robust group. The average total step count was significantly different and was approximately four times higher in the coaching period than in the self-management period (5,584,295.83 vs 1,289,084.66, *P*<.001). We found that participants in the “long-self” group who used the wearable device for the longest time showed increased body weight and body mass index by mean 0.65 (SD 1.317) and mean 0.097 (SD 0.513), respectively, compared with the other groups.

**Conclusions:**

Our “Smart Walk” program improved physical fitness, anthropometric measurements, and geriatric assessment categories in a small group of older adults in rural areas with limited resources for monitoring. Further validation through various rural public health centers and in a large number of rural older adults is required.

## Introduction

Unlike young adults, elderly individuals have different medical characteristics. Each older adult has a highly heterogeneous health status [1,2]. Even with definite illness, symptoms are frequently ambiguous and nonspecific in older adults [1]. Also, many frail older adults have diminished physiologic reservoirs and easily deteriorate regardless of their comorbidities [3]. Therefore, early detection and an appropriate preventive approach are crucial for older adults.

In recent decades, a frailty-based approach has been widely applied in communities having an older population [4]. Frailty is an age-related syndrome characterized by decreased physiologic reserve and increased vulnerability to stressors that lead to adverse health outcomes, such as disability, falls, institutionalization, and mortality [3]. Unlike the traditional comorbidity-based approach, a frailty-based strategy includes the concepts of an individualized approach, disability prevention, and enhancing quality of life regardless of age [5].

Many of the older adults living in the community show sedentary behavior. Reports have shown that >60% of community- dwelling older adults are sedentary [6]. Sedentary behavior is an important risk factor for cardiovascular disease [7], falls [8], and frailty [9] in older adults and is known to be associated with high mortality [10]. Therefore, preventing sedentary behavior and increasing physical activity among older adults are important in the public health care model.

However, community-dwelling older adults living in rural areas are in a less favorable environment for health care compared to those living in an urban environment. Healthcare facilities in rural areas are usually poor or have limited accessibility [11]. In addition, rural dwellers have a relatively lower socioeconomic status and education level and a higher prevalence of living alone, multimorbidity, frailty, and disability than urban older adults [12]. Thus, the role of a public health center is increasingly emphasized in rural areas with an optimized public health strategy. However, rural public health centers should also service relatively larger areas of dispersed older adults with insufficient resources, making it difficult to manage a cost-effective health care model. In addition, like several other Asian countries, South Korea has seen a more rapid older population growth in rural areas than in urban areas [13]. These greater burdens of aging-related health conditions and resource barriers for health care in rural areas facilitate the paradigm shift from disease management to health care and prevention.

The health benefits of wearable devices are known in the older population [14]. Accurately assessing the physical activity of older adults through interviews and examination requires considerable time and effort [15]. Wearable devices have been applied to monitor physical activity, falls, or behavior of community-dwelling older adults and have been applied to change the lifestyle and reduce metabolic risk of older adults with chronic diseases [16-19]. From the perspective of public health care, mobile health care services are in the limelight. Specifically, such mobile services have been shown to induce behavioral change by adding coaching or incentives to wearable devices [20]. Some wearable devices could be suitable upon adjusting for rural health resources. However, reports have shown that adherence is reduced to <10% when the benefit to the participants disappears [21]. Furthermore, these incentives can only be supported for a limited period. In rural areas, developing a wearable device-based health care program that can be operated with a small amount of resources and can encourage voluntary self-management after the end of the program is important.

Despite unfavorable conditions, public health centers in rural areas have several unique strengths for community studies [11]. Geographical isolation from private hospitals helps maximize the participation rate of the senior population within a short period, as well as the long-term retention rate [22,23]. We believed that a mobile health care service centered on a public health center is the most affordable, easy to access, and relatively costless method for health care of rural older adults. This study aimed to evaluate whether a simple mobile health care device and mobile-based intermittent coaching or self-management can increase the physical activity and health outcome of older adults in rural areas.

This study aimed to identify answers to the following three questions: (1) Can a wearable device improve physical activity and health outcomes of older adults in rural areas? (2) Are there differences in physical activity and health outcome improvement depending on frailty status? (3) Are there differences in wearable device adherence between coaching and self-management? To address the above questions, we carried out the “Smart Walk” program as a health care model using a wearable device to promote self-exercise among community-dwelling older adults managed by a community health center.

## Methods

### Study Design

We randomly selected older participants who had enrolled in the population-based, prospective cohort study of aging, the Aging Study of Pyeongchang Rural Area (ASPRA). The ASPRA cohort was established in October 2014 to determine the burden of frailty and geriatric syndromes in rural areas, understand the disparities between urban and rural older populations, and set the priority of public health interventions. The design and measurement protocol are described elsewhere [11,12]. Briefly, participants living in Pyeongchang rural area, located 180 kilometers east of Seoul, South Korea, were administered an annual comprehensive geriatric assessment including physical, mental, psychosocial, and frailty status, as well as medical conditions. The inclusion criteria were (1) being aged ≥65 years; (2) being registered in the National Healthcare Service; (3) being ambulatory with or without an assistive device; (4) living at home; and (5) being able to provide informed consent. Those who were living in a nursing home, hospitalized, or bed-ridden and receiving nursing-home-level care at the time of enrollment were excluded. To conduct this project, an academic-public health collaborative team was organized, and about 95% of eligible older adults in the study area were enrolled. The characteristics of ASPRA participants were similar to those of the Korean rural population represented in the Korea National Health and Nutrition Examination Survey [11].

### Frailty and Comprehensive Geriatric Assessment

In this study, we screened for potential participants using the Cardiovascular Health Study (CHS) frailty phenotype criteria, one of the most widely used assessment tools (Multimedia Appendix 1). For quantitative evaluation of frailty status, we used the frailty index suggested by Rockwood et al [24], which encompasses physical, cognitive, psychosocial, geriatric syndrome, disability, and underlying disease. Scores vary from 0 to 1; a higher score indicates a more severe frailty burden [23].

Trained nurses administered the comprehensive geriatric assessment using the following instruments: the International Physical Activity Questionnaire (IPAQ) short form, the Korean version of the Mini-Mental State Examination for cognitive function, the Korean version of the Center for Epidemiological Studies Depression scale for depressive mood, usual gait speed, the Mini Nutritional Assessment-Short Form (MNA-SF) score for malnutrition, multimorbidity, grip strength on dominant arm, the Short Physical Performance Battery score, and bioimpedance analysis using Inbody 620 (Inbody, Seoul, Korea). Detailed methods were described previously [11].

### Smart Walk Program

The “Smart Walk” program was a 13-month program conducted from March 2017 to March 2018, consisting of 6 months of coaching management, 1 month of rest, and 6 months of self-management. Initially, the health center staff provided a wearable device to the participants, helping with the installation of apps on mobile phones and mobile phone pairing. Participants established a “buddy” relationship with health center staff through the mobile app, which allowed them to monitor the activities and walks of each participant. If a problem with the connection between the mobile phone and wearable device appeared, the participants were asked to visit the health center to solve the problem.

Coaching was performed by 8 health center staff through notification messages of the wearable device. If no record of device use existed, weekly follow-up was performed. During the first 2 months, health center staff set all participants’ wearable device to a goal of 5000 steps per day, and in the 6th month of coaching period, 7000 steps were finally set as the daily target. This goal was reset such that the daily target was increased by 1000 steps every 2 months, and 7000 steps per day were finally required via wearable device in the 6th month of the coaching period. If no device use is observed or if the target step number is not reached, a push alarm is sent first through the app to encourage device use and exercise. After that, the health center staff checked the health status of the participant by phone or visit within a few days. Regarding education, health center staff did not instruct the participants on how to exercise unless the participants so requested.

An incentive was provided (including two group picnics, US $50 worth of nutritional supplements, and a wearable device worn by the participant) to encourage participants during the 6-month coaching period only, followed by a 7-month follow-up with monthly questionnaires and data logs from the wearable device. Coaching was performed by 8 health center staff through notification messages of the wearable device. If no record of device use existed, weekly follow-up was performed.

As no existing criteria for adherence to a wearable device was available, this study defined adherence as continuous use if the device was used for at least 1 week per month. This criterion refers to the follow-up at weekly intervals during the coaching period.

This study was approved by the Institutional Review Board of the Asan Medical Center (Institutional review board no. 2015-0673). We obtained a research study personal information agreement and exercise commitment letter from the study participants.

### Data Description

In this study, we collected three types of data: (1) data from the whole period the device was worn; (2) Comprehensive geriatric assessment before and after the coaching program; and (3) monthly questionnaire data during the follow-up period. The wearable device used in this study was a Xiao Mi band 2. We chose this device because it was the model with the lowest battery consumption (up to 3 weeks on a single charge). Only step count was used for analysis. All participants underwent comprehensive geriatric assessment that encompassed the assessment of cognitive and physical function, depression, nutrition, and body composition using bioimpedance analysis identical with the protocol of the ASPRA cohort and an additional 3-minute walk test before and after the coaching program [25]. Detailed measurements and definitions are described elsewhere [11]. A monthly assessment of geriatric conditions, such as frailty screening, falls, number of hospital visits, and weight loss, was administered by an experienced nurse based on a one-on-one telephone or face-to-face interview. The nurse also obtained information on demographic characteristics, living status, occupation, income, education level, chronic conditions, etc (detailed variables can be found in a study by Hee-Won Jung et al [11]).

### Data Analysis

**Figure 1:**
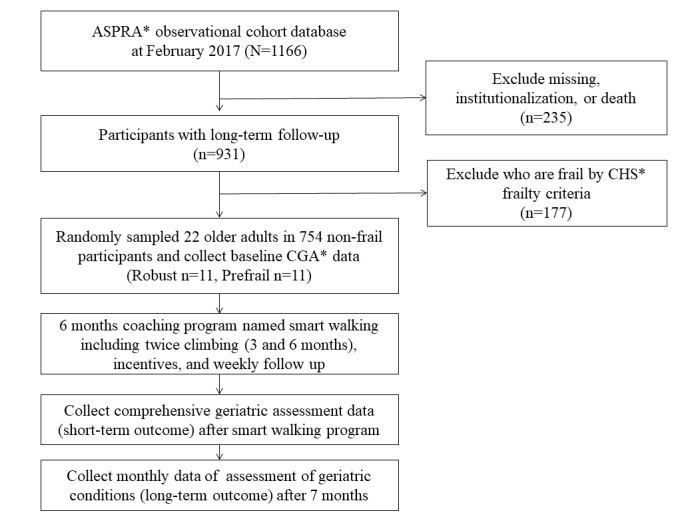
Recruitment of the Smart Walk program in the Aging Study of Pyeongchang Rural Area cohort. ASPRA: Aging Study of Pyeongchang Rural Area; CHS: Cardiovascular Health Study; CGA: Comprehensive Geriatric Assessment.

Among a total of 1166 participants who participated in the ASPRA cohort, we excluded 177 participants who were graded within the frail state (based on the CHS frailty criteria [26]) given that their participation in the “Smart Walk” program would be difficult (Figure 1). Of the 754 participants who were classified as robust or prefrail, we randomly selected 22 older adults (robust group=11 and prefrail group=11) to participate in the “Smart Walk” program.

For random selection, we assigned random numbers to participants of the robust and prefrail groups except for frail participants using the CHS frailty criteria. The opportunity to participate in this study was given in the order of users with small random numbers. Random numbers were assigned using R software version 3.3.1 (R Foundation, Vienna, Austria). Those who could not walk 100 meters without an assistive device were excluded.

Initial data analysis compared the differences in physical activity and health outcomes according to frailty status. Since the total number did not exceed 30, we performed a nonparametric Mann-Whitney-Wilcoxon test and Fisher test for mean and ratio evaluation, respectively. To confirm the coaching effects before and after the “Smart Walk” program, we also performed a nonparametric Wilcoxon signed-rank test on the pairs. The last analysis analyzed health outcomes according to wearable device adherence. According to adherence, the participants of the program were divided into the following three groups: (1) coaching only; (2) short-term self; and (3) long-term self. The coaching only group comprised persons who used the band only during the 6-month coaching program. During the 6-month follow-up period, those who used the band for 3 months were included in the short-term self group and those who used the band for 6 months were included in the long-term self group. The Kruskal-Wallis test was performed to evaluate differences among the three groups, and all pairwise analyses were performed using the Mann-Whitney-Wilcoxon and Fisher tests for mean and ratio, respectively. All reported *P* values were two-sided, and *P* values <.05 were considered significant. Data analyses were conducted using R software, version 3.3.1 (R Foundation, Vienna, Austria).

## Results

### Overall Characteristics

From March 2017 to March 2018, 22 older adults participated in the Smart Walk program (robust group: n=11, male: 8/11; prefrail group: n=11, male: 6/11). The two groups were similar in most of the variables, except for age, frailty index, and Short Physical Performance Battery score associated with the CHS frailty criteria (Table 1). Although not statistically significant, the variables of living alone, including the risk of malnutrition and falls in the past year, were higher in the prefrail group. None of the participants had low income and received national medical aid.

**Table 1:** Overall characteristics of the Smart Walk program participants by the Cardiovascular Health Study frailty index.

Variables	Robust (n=11)	Prefrail (n=11)	Total (N=22)	*P* value
Age, mean (SD)	68.636 (1.85)	72.546 (4.298)	70.591 (3.801)	.03
Gender (male), n (%)	8 (73)	6 (55)	14 (64)	.66
Education level, mean (SD)	11.546 (4.22)	10.455 (5.3)	11 (4.711)	.66
Living alone, %	0 (0)	2 (18)	2 (9)	.48
Frailty index, mean (SD)	0.075 (0.04)	0.171 (0.085)	0.123 (0.083)	.005
Multimorbidity, %	0.099 (0.11)	0.141 (0.094)	0.12 (0.106)	.16
Cognition: MMSE^a^ score, mean (SD)	28.818 (0.60)	27.909 (1.814)	28.364 (1.399)	.25
Mood: CES-D^b^ score, mean (SD)	2.091 (2.73)	4.818 (4.262)	3.455 (3.764)	.09
Body mass index, mean (SD)	24.269 (2.74)	25.416 (3.478)	24.842 (3.115)	.95
At risk of malnutrition: MNA-SF^c^, %	1 (9)	2 (18)	3 (14)	>.99
Short Physical Performance Battery score, mean (SD)	11.818 (0.40)	10.546 (0.934)	11.182 (0.958)	<.001
Dominant grip strength, mean (SD)	35.673 (9.01)	28.309 (7.99)	31.991 (9.125)	.13
Fall in the past year, %	0 (0)	3 (27)	3 (14)	.21

^a^MMSE: Mini-Mental State Examination.

^b^CES-D: Center for Epidemiological Studies Depression.

^c^MNA-SF: Mini Nutritional Assessment-Short Form.

### Comparison of Health Improvement During the Coaching Program According to Frailty Status

We analyzed health improvement according to frailty status through coaching by managers of the public health center for the first 6 months of the Smart Walk program. No statistically significant difference was observed in the robust group, but the prefrail group showed significant improvement in usual gait speed (mean 0.73 [SD 0.11] vs mean 0.96 [SD 0.27], *P*=.02), IPAQ scores kcal (mean 2790.36 [SD 2224.62] vs mean 7589.72 [SD 4452.52], *P*=.01) [27], and European Quality of Life-5 Dimensions score (mean 0.84 [SD 0.07] vs mean 0.90 [SD 0.07], *P*=.02) [28]. In the total group, physical fitness, anthropometric measurements, and geriatric assessment categories, such as usual gait speed (mean 0.85 [SD 0.21] vs mean 1.02 [SD 0.27], *P*=.003), IPAQ score in kcal (mean 3013.63 [SD 2387.08] vs mean 7868.5 [SD 6250.56], *P*=.001), body mass index (BMI; mean 24.84 [SD 3.11] vs mean 24.52 [SD 3.36], *P*=.02), and total fat mass (mean 18.85 [SD 6.15] vs mean 17.82 [SD 6.41], *P*=.01) were significantly improved (Multimedia Appendix 2).

### Comparison of Wearable Device Adherence During Coaching and Self-Management

**Figure 2:**
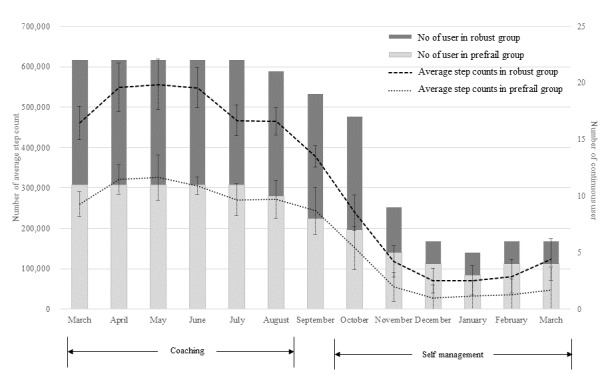
Average step count and number of continuous users in the robust and prefrail groups by month.

A large difference in the proportion of continuous wearable device users was found between the coaching and self-management periods (average: 21.83 vs 8.16 persons, *P*<.001; see Figure 2). Figure 2 shows the average step count and number of continuous users in the robust and prefrail groups by month. The histogram plot indicates continuous users per month, and the line plot indicates the average total step count per month. The error bar of the line plot represents the SD of the average step count. In particular, the total step average was significantly different between the two periods; the total step average of the coaching period was about four times higher than that of the self-management period (5,584,295.83 vs 1,289,084.66, *P*<.001). The average monthly steps of the robust group and the prefrail group also differed markedly during the coaching period, while both were lower during the self-management period.

Of the 22 users, 5 (robust: 1 and prefrail: 4) people participated only in the coaching period (from March to September), and 11 (robust: 8 and prefrail: 3) people participated in the short self-management period (from March to December). The long self-management period (March 17 to March 18) included 6 (robust: 2 and prefrail: 4) people. An unusual finding was that prefrail persons used the wearable device longer than robust persons, but the average step number per month in the prefrail group was half that in the robust group.

**Figure 3:**
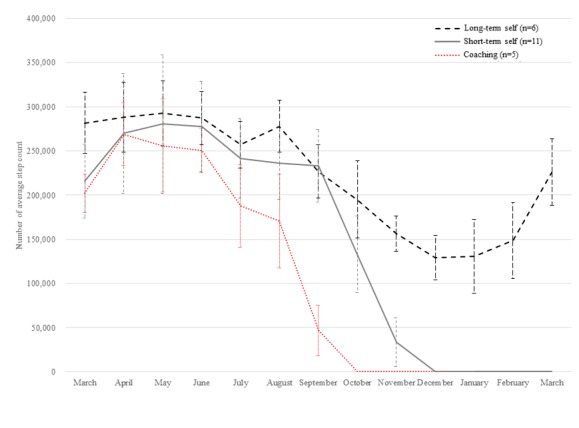
Monthly average step count by group of continuous use of the wearable device.

Figure 3 compares the monthly average number of steps and SD between the three groups (coaching, short-term self, and long-term self). The long-term self group and the coaching group differed significantly (average: 106,309.15 vs 222,725.73 steps, *P=*.02). The short-term self group was similar to the coaching group at the beginning of the program, but it was more similar to the long-self group in the middle term.

**Table 2:** Comparison of health improvement according to adherence type of the wearable device.

Characteristic	Coaching (n=4), mean (SD)	Short (n=10), mean (SD)	Long (n=6), mean (SD)	*P* value^a^
Number of falls	0.25 (0.5)	0.4 (1.265)	0 (0)	.49
Number of outpatient days	10.5 (10.472)	10 (6.864)	10.5 (4.722)	.10
Number of admission days	0 (0)	0 (0)	0 (0)	—^b^
Number of emergency room days	0.25 (0.5)	0 (0)	0 (0)	.14
Weight, kg	−1.325 (1.162)	−0.65 (1.824)	0.65 (1.317)	.07
Body mass index, kg/m^2^	−0.416 (0.446)	−1.097 (3.373)	0.097 (0.513)	.30
Mini Nutritional Assessment score	0.25 (2.062)	−0.2 (1.687)	0.333 (0.516)	.66
K-frail score	0 (1.414)	0.3 (1.059)	0.167 (0.408)	.60

^a^The Krukal-Wallis test, a nonparametric test of ≥3 groups, was performed.

^b^Not applicable.

### Health Improvement Comparison According to Wearable Device Adherence

During the research period, 2 participants were dropped. According to the CHS frailty criteria, both were in the robust group. The dropped participants refused geriatric assessment 4 months after the coaching period. However, they showed >90% adherence throughout the 6-month coaching period and were classified into the coaching group.

In the long-term self group, not a single fall was observed during the entire 13-month period, while an average of 0.25 and 0.4 falls occurred in the coaching and short-term self groups, respectively (Table 2). The average number of outpatient days was ≥10 in all three groups, with little difference between groups. No hospital admission occurred in all three groups. Emergency room visit was reported in only one case in the coaching group, wherein a food poisoning event occurred in a 72-year-old man at the end of the self-management period. Weight decreased by −1.325 (SD 1.824) and −0.65 (SD 1.317) in coaching and short-term self groups, respectively, and increased by 0.65 (SD 1.317) in the long-term self group. Similarly, BMI also increased in the long-term self group to 0.097 (SD 0.446) and decreased to −0.416 (SD 0.446) in the coaching group and to −1.097 (SD 3.373) in the short-term self group. MNA values decreased in the short-term self group but increased in the other two groups. No significant difference was found between all pairwise two-group comparisons (Multimedia Appendix 3).

## Discussion

### Principal Findings

This feasibility study showed that the wearable device-based intervention had a significant effect on physical performance (usual gait speed and IPAQ score) and anthropometric measurements (BMI and total fat mass) in rural older adults. In particular, usual gait speed (*P*=.02), IPAQ score (*P*=.01), and European Quality of Life-5 Dimensions-3L score (*P*=.02) were significantly improved in the prefrail group compared with the robust group. In addition, the compliance of wearable devices in this study confirmed a pattern of persistent use for ≥12 months by approximately 30% users (6/22 older adults) compared with the 10% 1-year continuous use rate in other studies [21]. The fact that 4 of the 6 users in the long-term self-management group belonged to the prefrail group supports the rationale that health concerns are among the factors that increase adherence to wearable devices. Also, we found that the long-term self group, which used the wearable device for the longest time, showed a 0.65 (SD 1.317) and 0.097 (SD 0.513) increase in body weight and BMI, respectively, compared with the other groups. Decreased body weight in older adults is not a positive indicator for health outcomes due to increasing loss of muscle mass [28]. In this respect, we improved the health outcomes of the older population through the long-term use of wearable devices. Our findings match those of previous studies reporting that individualized programs and self-management techniques could enhance physical activity adherence among older adults [29-31].

Our positive results may be explained by two factors. First, in order to maintain high adherence, we collaborated with public health centers to reduce the cost of managing and encouraging older adults and chose a wearable device with low management costs. Lewis et al reported that wearable-only interventions tend to produce only a modest effect on improving physical activity behavior [32]. Therefore, we decided to not only select a wearable device but also add human intervention via collaboration with the public health center. The burden of checking on the participants via smartphone every week and sending a message to those with poor exercise patterns did not exceed 5% of the weekly working time of a health center worker, even in a manpower-restricted rural public health center. When selecting the appropriate device for rural older adults, features like simplicity, ease of use, affordability, and long battery life (>3 weeks from a single charge) helped achieve a higher adherence.

Second, after comparing coaching and self-management components, older people demonstrated that the long-term use of wearable devices can be increased as needed. Many studies on the strengths of wearable devices are available, but several studies have still not maintained sustainable use and therefore have poor health outcomes [21,33]. However, the higher long-term use in the less healthy prefrail older adults in this study may lead to a new perspective on adherence to wearable devices. In other words, if long-term management is performed for users who need health care, not the general public, the low adherence to wearable devices can be improved. In this study, the long-term self group using long-term wearable devices showed improved health outcomes in terms of nutrition (MNA score), physical activity (number of steps), and anthropometric measurements (weight and BMI).

### Limitations

The main limitation of this study is that it did not have many participants. The first goal of this study was to examine the possibility of using wearable devices for older people. Hence, a feasibility study utilizing a small number of individuals with different frailty levels rather than a large number of users was required. As a result, we verified the high level of adherence to and health outcomes of wearable devices in this study. Based on the results of this study, further studies should plan to include many older adults with various frailty levels. The second limitation is that this study excluded frail older adults. The targeted daily step count of the wearable device was set by the researcher to increase by 1000 steps every 2 months and finally reach 7000 steps. Thus, frail older adults were not suitable for this study and were excluded, which could worsen the conditions of the frail adults. Further studies, however, are required to improve the health outcomes of these frail older adults.

## Appendix

Multimedia Appendix 1 The 34 components of frailty index.

Multimedia Appendix 2 Comparison of health improvement according to the Cardiovascular Health Study frailty index with the coaching program.

Multimedia Appendix 3 All pair wised comparison of health improvement according to the adherence type of the wearable device.

## Abbreviations

ASPRA: Aging Study of Pyeongchang Rural Area
BMI: body mass index
CHS: Cardiovascular Health Study
IPAQ: International Physical Activity Questionnaire
MNA-SF: Mini Nutritional Assessment-Short Form
