# Supplementary material for: Impact of a Wearable Device-Based Walking Programs in Rural Older Adults on Physical Activity and Health Outcomes: Cohort Study
Source: JMIR Mhealth Uhealth. 2018 Nov 21;6(11):e11335. doi: 10.2196/11335 (PMC6282012; doi:10.2196/11335)
Supplement: Multimedia Appendix 1 [file mhealth_v6i11e11335_app1.pdf]

Multimedia Appendix 1. The 34 components of frailty index.

- Changes in everyday activities
- Problems getting dressed
- Problems with bathing
- Problems carrying out personal grooming
- Urinary incontinence
- Toileting problems
- Gastrointestinal problems
- Problems cooking
- Problems going out alone
- Impaired mobility
- Musculoskeletal problems
- Bradykinesia of the limbs
- Poor muscle tone in limbs
- Poor limb coordination
- Poor coordination, trunk
- Poor standing posture
- Irregular gait pattern
- Falls
- Mood problems
- Tiredness all the time
- Depression
- Memory changes
- Changes in general mental functioning
- History relevant to cognitive impairment or loss
- History of stroke
- History of diabetes mellitus
- Arterial hypertension
- Cardiac problems
- Myocardial infarction
- Congestive heart failure
- Lung problems
- Respiratory problems
- Malignant disease
- Other medical history
