# Supplementary material for: Impact of a Wearable Device-Based Walking Programs in Rural Older Adults on Physical Activity and Health Outcomes: Cohort Study
Source: JMIR Mhealth Uhealth. 2018 Nov 21;6(11):e11335. doi: 10.2196/11335 (PMC6282012; doi:10.2196/11335)
Supplement: Multimedia Appendix 2 [file mhealth_v6i11e11335_app2.pdf]

Multimedia Appendix 2. Comparison of health improvement according to the Cardiovascular Health Study frailty index with the coaching program.

Health improvement characteristic	Robust (n=11)			Prefrail (n=11)			Total (N=22)		
	Pre	Post	*P*	Pre	Post	*P*	Pre	Post	*P*
**Physical performance, mean (SD)**									
3-minute walk test, meters	249.18 (76.59)	266.00 (50.40)	0.24	236.81 (110.37)	252 (80.06)	.39	243 (92.92)	259.00 (65.67)	.14
Maximal grip strength in dominant arm, kg	35.67 (9.01)	35.06 (7.52)	0.36	28.30 (7.99)	29.25 (7.00)	.91	31.99 (9.12)	32.15 (7.69)	.65
Usual gait speed, sec	0.97 (0.21)	1.08 (0.26)	0.12	0.73 (0.11)	0.96 (0.27)	*.02*	0.85 (0.21)	1.02 (0.27)	.003
Chairstand, sec	8.06 (1.98)	7.34 (2.01)	0.14	8.92 (2.56)	8.58 (1.64)	.89	8.49 (2.27)	7.96 (1.90)	.33
IPAQ[a] kcal, MET-min/wk	3236.90 (2628.22)	8147.27 (7877.19)	0.05	2790.36 (2224.62)	7589.72 (4452.52)	*.01*	3013.63 (2387.08)	7868.5 (6250.56)	.001
**Anthropometric measurements, mean (SD)**									
Body mass index, kg/m^2	24.26 (2.74)	23.83 (2.90)	0.08	25.41 (3.47)	25.21 (3.77)	.24	24.84 (3.11)	24.52 (3.36)	.02
Skeletal Muscle Index adjusted by height^2 [kg/m^2]	7.38 (0.76)	7.47 (0.74)	0.17	6.84 (0.78)	6.89 (0.83)	0.41	7.11 (0.80)	7.18 (0.82)	.11
Total fat mass [kg]	16.82 (5.27)	15.69 (5.04)	0.06	20.88 (6.53)	19.95 (7.14)	.10	18.85 (6.15)	17.82 (6.41)	.01
**Geriatric assessment, mean (SD)**									
CES-D[b] score	2.09 (2.73)	4.09 (4.46)	0.33	4.81 (4.26)	6.81 (6.22)	.34	3.45 (3.76)	5.45 (5.46)	.13
EuroQol 5D-3L[c] score	0.92 (0.02)	0.92 (0.05)	1	0.84 (0.07)	0.90 (0.07)	.02	0.88 (0.06)	0.91 (0.06)	.05
MMSE[d] score	28.81 (0.60)	28.81 (0.87)	1	27.90 (1.81)	28.54 (2.01)	.10	28.36 (1.39)	28.68 (1.52)	.17

[a]IPAQ: International Physical Activity Questionnaire.

[b]CES-D: Center for Epidemiological Studies Depression.

[c]EuroQol 5D-3L: European Quality of Life-5 Dimensions.

[d]MMSE: Mini-Mental State Examination.
