# Supplementary material for: Impact of a Wearable Device-Based Walking Programs in Rural Older Adults on Physical Activity and Health Outcomes: Cohort Study
Source: JMIR Mhealth Uhealth. 2018 Nov 21;6(11):e11335. doi: 10.2196/11335 (PMC6282012; doi:10.2196/11335)
Supplement: Multimedia Appendix 3 [file mhealth_v6i11e11335_app3.pdf]

**Multimedia Appendix 3.** All pair wised comparison of health improvement according to the adherence type of the wearable device.

| | Long-self (N=6) | Short-self (N=10) | p-value | Coaching (N=4) | Long-self (N=6) | p-value | Coaching (N=4) | Short-self (N=10) | p-value |
|---|---|---|---|---|---|---|---|---|---|
| Number of falls | 0±0 | 0.4±1.265 | 0.519 | 0.25±0.5 | 0±0 | 0.307 | 0.25±0.5 | 0.4±1.265 | 0.643 |
| Number of outpatient days | 10.5±4.722 | 10±6.864 | 0.87 | 10.5±10.472 | 10.5±4.722 | 0.915 | 10.5±10.472 | 10±6.864 | 0.944 |
| Number of admission days | 0±0 | 0±0 | NaN | 0±0 | 0±0 | NaN | 0±0 | 0±0 | NaN |
| Number of ER days | 0±0 | 0±0 | NaN | 0.25±0.5 | 0±0 | 0.307 | 0.25±0.5 | 0±0 | 0.155 |
| Weight | 0.65±1.317 | -0.65±1.824 | 0.057 | -1.325±1.162 | 0.65±1.317 | 0.054 | -1.325±1.162 | -0.65±1.824 | 0.671 |
| KFRAIL score | 0.167±0.408 | 0.3±1.059 | 0.943 | 0±1.414 | 0.167±0.408 | 0.469 | 0±1.414 | 0.3±1.059 | 0.431 |
| Body Mass Index | 0.097±0.513 | -1.097±3.373 | 0.313 | -0.416±0.446 | 0.097±0.513 | 0.114 | -0.416±0.446 | -1.097±3.373 | 0.839 |
| MNA score | 0.333±0.516 | -0.2±1.687 | 0.392 | 0.25±2.062 | 0.333±0.516 | 0.718 | 0.25±2.062 | -0.2±1.687 | 0.827 |
